# Microbial succession from a subsequent secondary death event following mass mortality

**DOI:** 10.1186/s12866-020-01969-3

**Published:** 2020-10-13

**Authors:** Lindsay Harrison, Emilia Kooienga, Cori Speights, Jeffery Tomberlin, Marcus Lashley, Brandon Barton, Heather Jordan

**Affiliations:** 1 Department of Biological Sciences, Mississippi State University, PO Box GY, Mississippi State, MS 39762 USA; 2 Department of Entomology, Texas A&M University, Minnie Bell Heep Center, Suite 412, College Station, TX 77843 USA; 3 Department of Wildlife, Fisheries and Aquaculture, Mississippi State University, Box 9680, Mississippi State, MS 39762 USA

**Keywords:** Microbial succession, Decomposition, Mass mortality

## Abstract

**Background:**

Each death event can be characterized by its associated microbes – a living community of bacteria composed of carcass, soil, and insect-introduced bacterial species – a necrobiome. With the possibility for close succession of these death events, it may be beneficial to characterize how the magnitude of an initial death event may impact the decomposition and necrobiomes of subsequent death events in close proximity. In this paper we hope to characterize the microbial communities associated with a proximate subsequent death event, and distinguish any changes within those communities based on the magnitude of an initial death event and the biomass of preexisting carcass (es) undergoing decomposition. For this experiment, 6 feral swine carcasses in containers were placed in the vicinity of preexisting and ongoing carcass decomposition at sites of three different scales of decomposing carcass biomass. Swab samples were collected from the skin and eye sockets of the container pigs and subjected to 16 s rRNA sequencing and OTU assignment.

**Results:**

PERMANOVA analysis of the bacterial taxa showed that there was no significant difference in the bacterial communities based on initial mortality event biomass size, but we did see a change in the bacterial communities over time, and slight differences between the skin and ocular cavity communities. Even without soil input, necrobiome communities can change rapidly. Further characterization of the bacterial necrobiome included utilization of the Random Forest algorithm to identify the most important predictors for time of decomposition. Sample sets were also scanned for notable human and swine-associated pathogens.

**Conclusions:**

The applications from this study are many, ranging from establishing the environmental impacts of mass mortality events to understanding the importance of scavenger, and scavenger microbial community input on decomposition.

## Background

It is widely known that decomposition rate is influenced by temperature, burial, and access by invertebrate and vertebrate scavengers [1]. However, the effect of nearby decomposition events and the pre-existing presence of vertebrate scavengers, invertebrates, and microbes has not been extensively investigated. Also, while, an initial influx of decomposers such as blow fly (Diptera: Calliphoridae) larvae may quickly reduce a *single* corpse [1], decomposition rates will likely be impacted as biomass increases. With larger decomposing biomass there is also a greater capacity to support a larger insect population. This could have an impact on subsequent mortality events as early decomposers may arrive more quickly, and transfer microbial communities from an already established necrobiome [2]. Vertebrate scavengers may also change behavior in response to a large-biomass death event due to an increase in availability and quality of nutrient resources [3]; and due to legacy effects, may access carrion from a subsequent death event more quickly. Reduced competition due to abundant resources and proximity of subsequent events could affect arrival times, migration patterns, and decomposition rates.

Microbial activity in, on, and around carcasses is a recently recognized variable which can affect decomposition rates [4, 5]. Microbes are a driving factor in ecosystem processes including decomposition, pathogenic spread, and nutrient recycling. Microbes are often specialized to certain substrates, and many have the capacity to break down organic matter [6]. Several studies have demonstrated the impact of soil communities as well as host-associated communities on decomposition rates and succession [7, 8]. Lauber, et al. conducted a study to analyze soil microbial communities driving decomposition processes and found that the presence of soil microbial communities significantly increase carrion decomposition rates [9]. However, the composition of soil microbial communities versus carcass-associated communities was not clearly differentiated in their data set. Furthermore, few studies have differentiated between microbial succession of hosts’ internal versus external microbial communities, in the absence of the soil microbiome.

We posit that hosts’ microbial diversity plays a key role in maintaining ecosystem multifunctionality by supporting processes such as decomposition, which allow transfer of matter and energy between other microbial communities and to higher trophic levels (such as soil or invertebrate communities, respectively). Thus, characterizing host microbial communities will lead to a better understanding of their impact on decomposition, for systems-level ecological prediction of diversity-disturbance relationships.

Insects also serve an important functional role during decomposition, and their importance in estimating post-mortem interval in forensic investigations is well established [10, 11]. As insects are among the first colonizers of a cadaver, they likely play an important role in establishing an initial carcass-specific microbiome and affect the succession of a carcass’s microbial community structure as decomposition progresses. If the initial colonizing population is one that has already been inoculated with necrobiome bacteria from an established mortality event, this may have an influence on the microbial succession of a carcass from a subsequent death event.

Another important question regarding carcass decomposition is whether or not they may be contributing to the increased spread of zoonotic, inter- or intra-species diseases. Very little is known regarding persistence of potential pathogens, transfer potential following host death, or whether this may be amplified with increasing biomass.

**Fig. 1:**
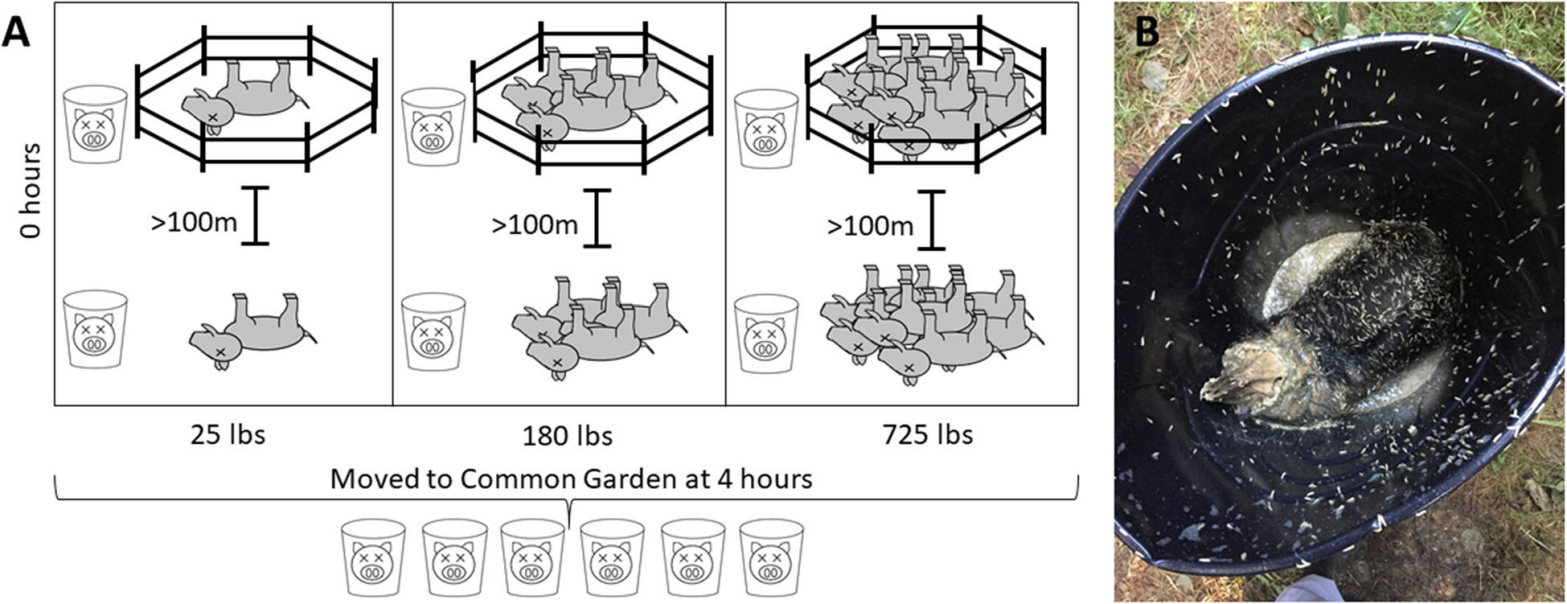
**a** Schematic of initial and secondary mortality event placement. The initial mortality event was made up of previously placed swine carcasses in increasing biomass at differing sites. Weights refer to the biomass of the initially placed swine biomass that is representing the initial death event. Fenced and unfenced replicates of initial decomposition sites of increasing biomass were constructed at least 100 m apart. Swine in the buckets were placed within 3 m of each initial mortality event site. After transport to the common garden, buckets were placed ~ 1 m apart. **b** Feral swine covered with insect larvae within enclosed bucket at the 4D timepoint. Insect larvae were allowed access to carcass for 4H at the initial mortality event sites. Swine were then covered in mesh and transported to a common garden

For this study, we placed individual swine into containers within the decomposition island of preexisting and ongoing study sites measuring decomposition and ecological effects among carcasses with increasing biomass (Fig. 1, [12] and unpublished data). The objective of this study was to characterize microbial succession and other ecological effects from a subsequent mortality event that occurs in the same location (or nearby but still within the first decomposition island). We did so in such a way that soil microbial communities and vertebrate scavengers and their associated microbiomes were excluded, while still allowing invertebrate access. This allowed us to investigate the succession of the microbial community from the swine microbiome and from microbial communities from invertebrates as early stages of decomposition progressed.

We hypothesized that the biomass from pre-existing decomposing carcasses could affect the microbial colonization and community richness and abundance within subsequent death events. Furthermore, we predicted that invertebrate access, but soil exclusion from the decomposition island, would also affect the richness of microbes expected and give us a better idea of which microbes are transferred from the initial carcass. Finally, we predicted that skin and ocular cavity (used to serve as a more invasive sampling location) microbial communities would differ in microbial richness and abundance, and that temporal changes in microbial communities would differ between the two tissue types.

## Results

There were no significant differences in beta diversity of microbial communities between the fenced and unfenced treatment PERMANOVA (F_1_ = 1.26, *p* = 0.25), nor between swine placed at different initial mass mortality event biomasses (F_1_ = 0.50, *p* = 0.78), and this was further supported by a linear regression (R^2^ = 0.001, *p* = 0.84).

**Fig. 2:**
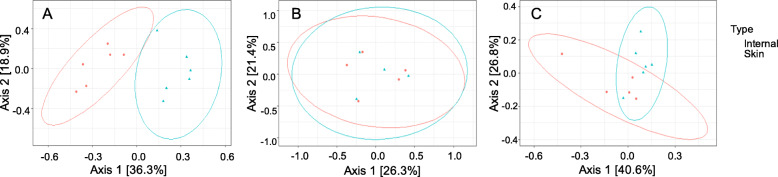
Principle component analysis plots of skin and Internal samples using Bray-Curtis distances at 0H (**a**, *p* = 0.003), 3H (**b**, *p* = 0.995), and 4D (**c**, *p* = 0.037) timepoints

**Fig. 3:**
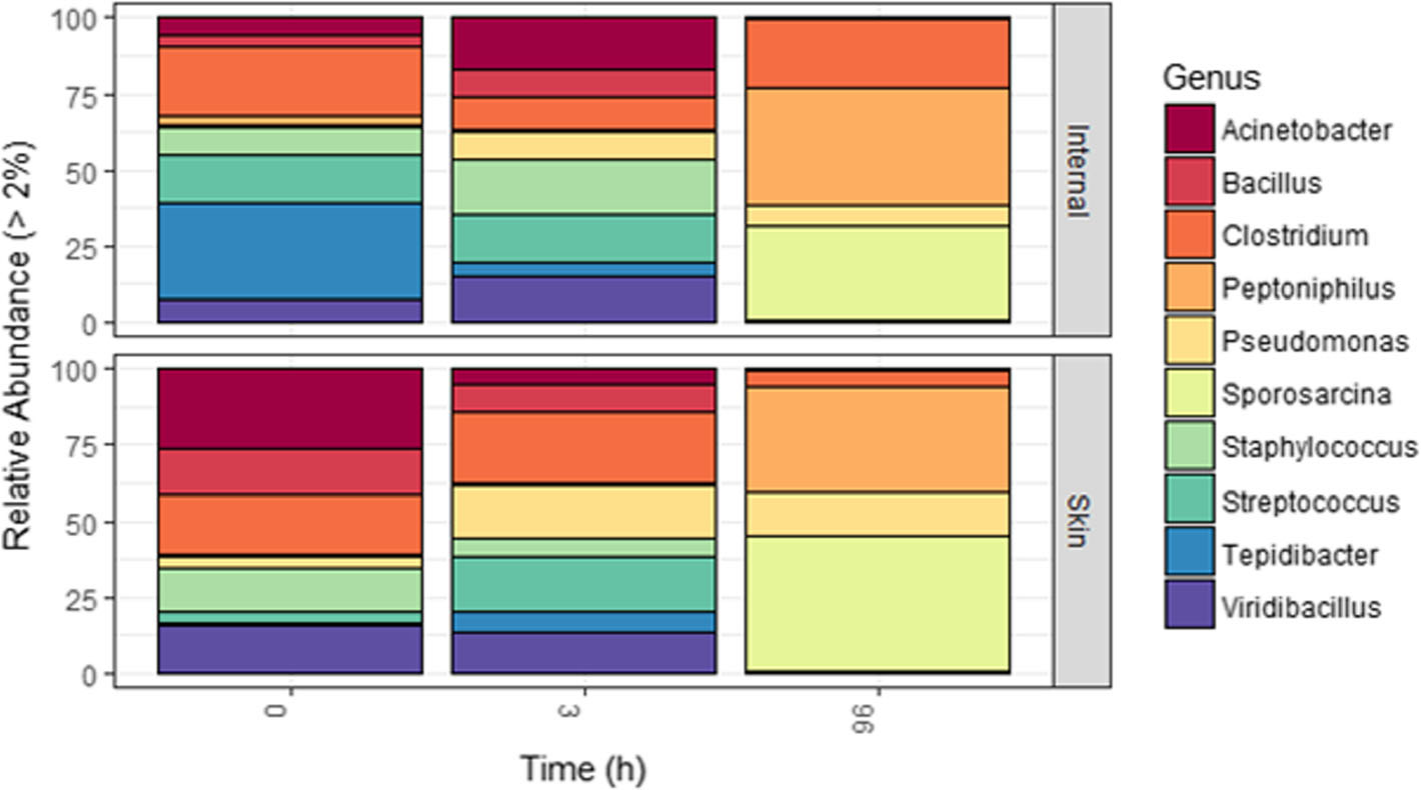
Genus-level relative abundance for skin and internal samples for each timepoint sampled. OTUs with less than 2% abundance were excluded

Principle component analyses using Bray-Curtis distances revealed a significant difference in Genus-level microbial diversity between skin and ocular cavity samples at 0 h (*p* = 0.003, Fig. 2a). This initial difference at 0 h was noted for all swine across biomass and fence-type plots with *Clostridium* and *Staphylococcus* being the two prominent unifying taxa (Fig. 3). A large proportion of *Tepidibacter* (17%) and *Clostridium* (14.5%) were identified in the ocular cavity samples, and skin samples showed a high number of *Viridibacillus* (8.8%) and *Acinetobacter* (8.5%) taxa. The significant difference in diversity between skin and ocular cavity samples was not present at the 3 h timepoint (*p* = 0.995, Fig. 2b) but was again significant at the 4 day timepoint (*p* = 0.037, Fig. 2c). At the 4 day timepoint, skin and ocular cavity samples had similar percentages of genera *Sporosarcina* and *Peptioniphilus*, but differences in *Clostridium* species (17% ocular cavity, 4% skin) (Fig. 3).

**Fig. 4:**
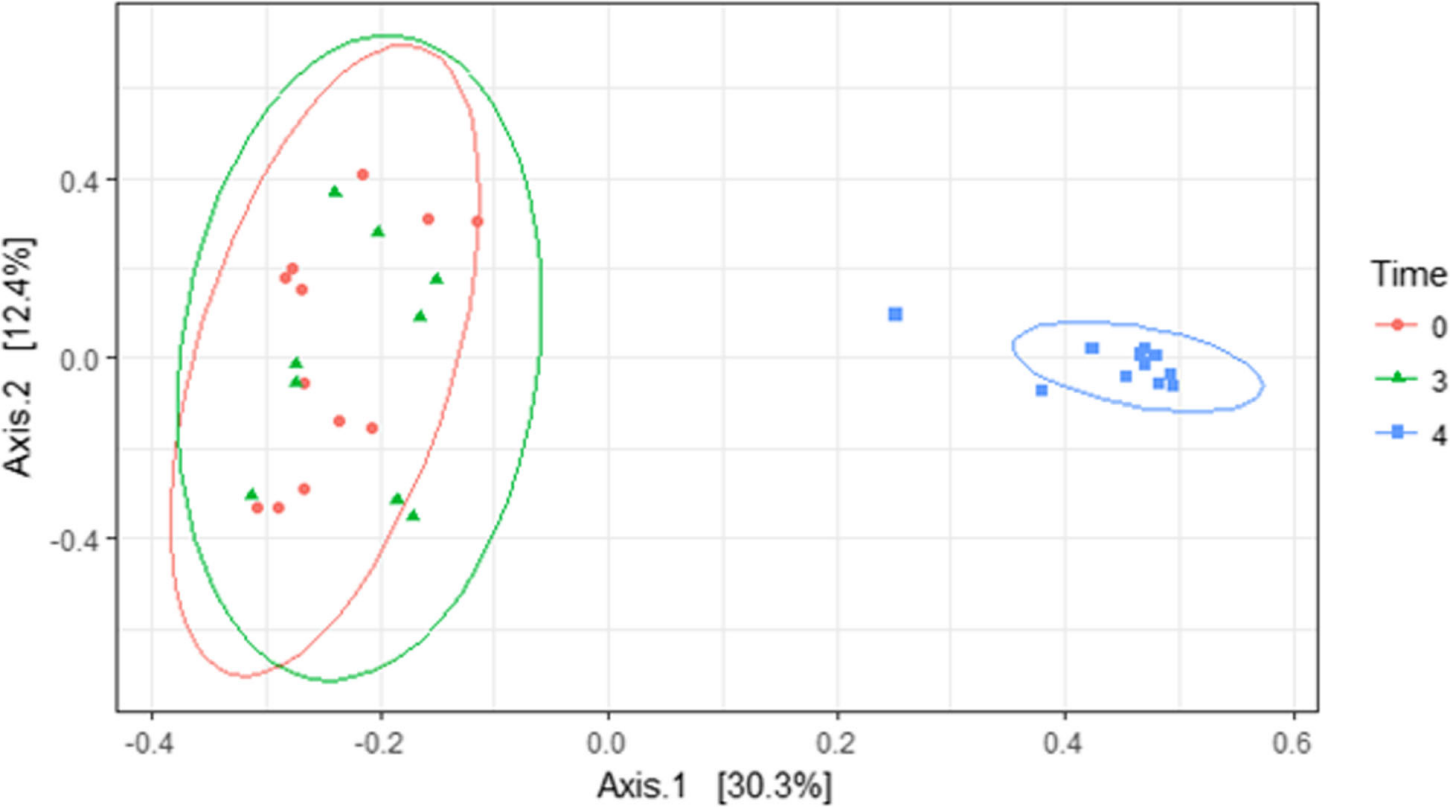
PCoA plot comparing overall community diversity at three timepoints using Bray-Curtis distances. Significance was found between 0 h and 3 h, and 4 day timepoints (*p* < 0.01)

**Fig. 5:**
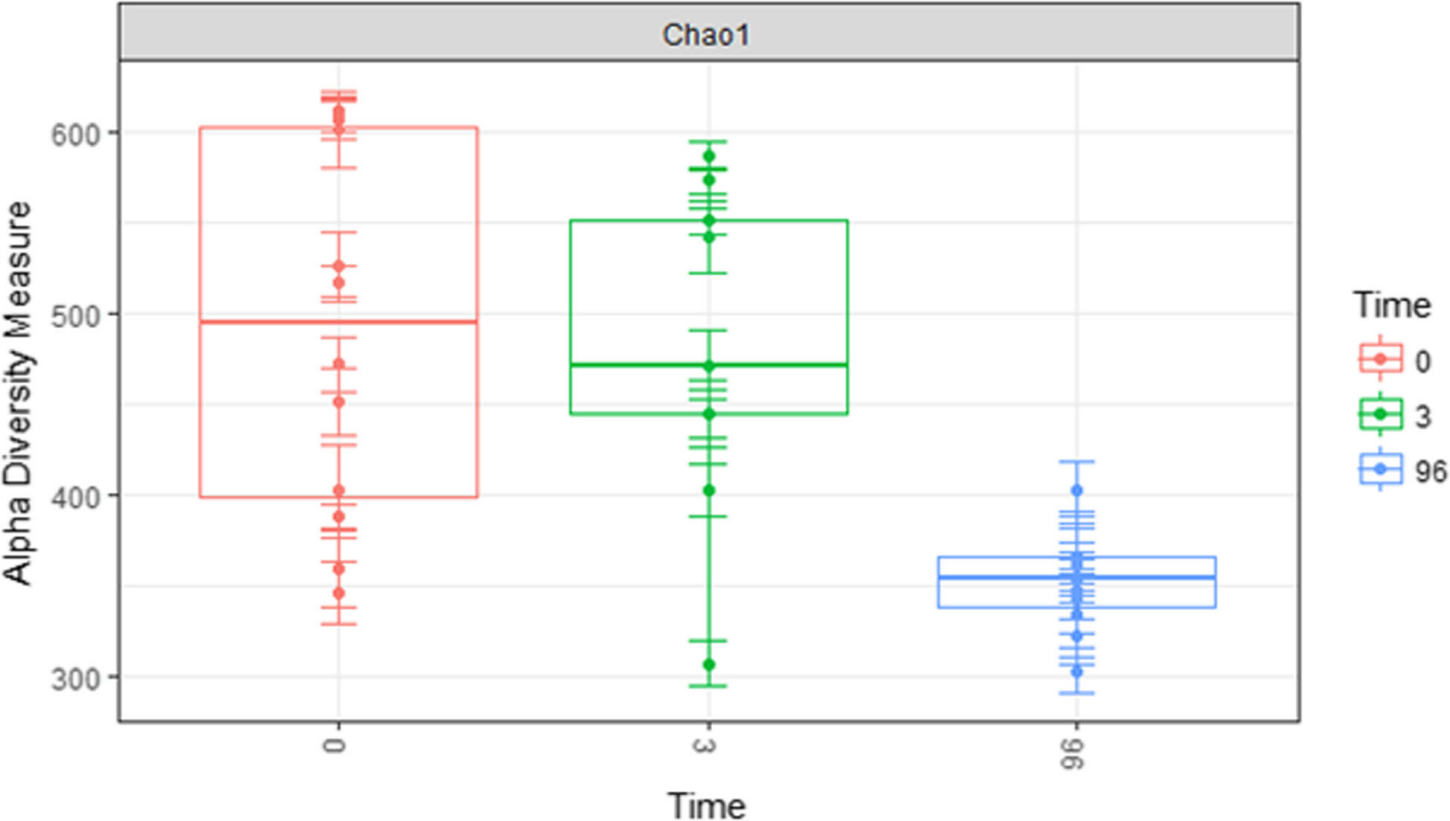
Alpha diversity of combined skin and internal communities by timepoint using Chao index from data rarefied to the lowest number of OTUs. A decreasing trend in diversity was observed

**Fig. 6:**
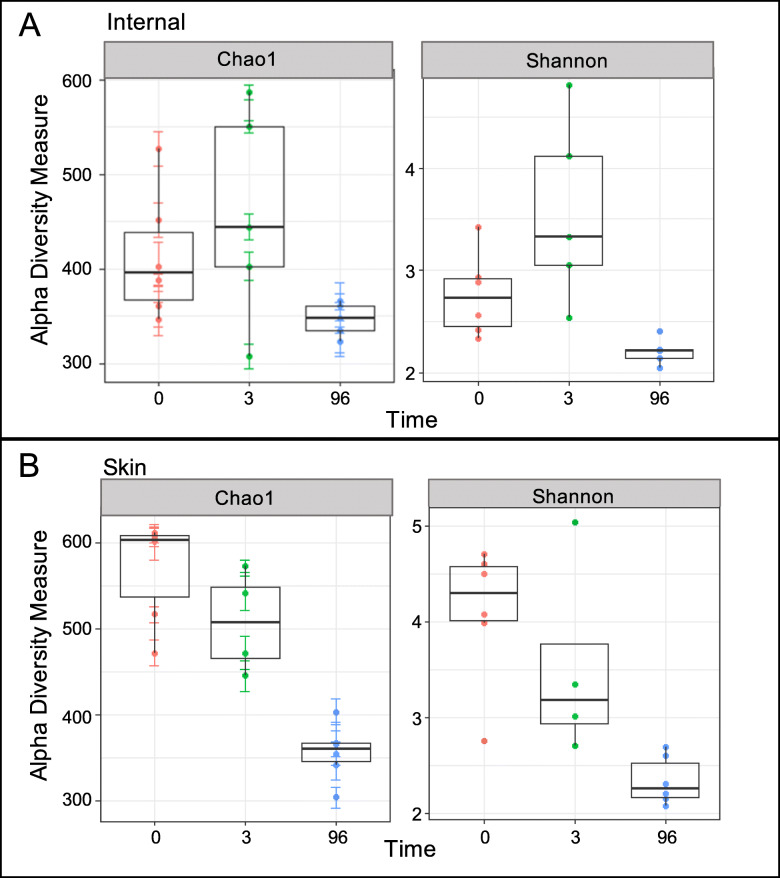
Alpha diversity using Chao1 and Shannon indices for skin (**a**) and internal (**b**) communities across three timepoints. Diversity showed a decreasing trend over time using both indices

A significant difference in total microbial diversity from combined skin and ocular cavity communities was detectable between the initial (0 h and 3 h) samples and the samples 4 days post-placement, but no difference was detected within the first 3 h (Table 1, Fig. 4). Diversity significantly decreased between the 0 h and 3 h samples and the 4 day samples (*p* = 0.001) (Fig. 5). This result was consistent for overall community diversity (Fig. 5), and also when skin and ocular cavity samples were separated (Fig. 6).

**Table 1:** Results of PERMANOVA analyses for each of the three timepoints using 999 permutations and rarefied data. Degrees of freedom was 1 for each test. A *p* value < 0.05 was considered significant

	Time 0		Time 3Hrs		Time 4 Days	
	F Stat.	P	F Stat.	P	F Stat.	P
Swab Type	4.27	0.003*	0.35	0.998	2.09	0.022*
Biomass	0.94	0.497	1.08	0.357	0.31	0.967
Fencing	1.10	0.315	1.57	0.120	1.27	0.239
Type*Biomass	0.74	0.700	1.25	0.177	2.22	0.061
Type*Fencing	1.03	0.390	1.40	0.137	0.72	0.594
Biomass*Fencing	0.89	0.527	1.05	0.378	1.35	0.245

*denotes a significant *p* value

The results of machine learning algorithm analyses identified a list of the top ten candidate taxa that differentiated the initial (0 h and 3 h) and 4 day timepoints (Table 2). *Sporosarcina*, *Peptoniphilus* and *Ignatzchineria* notably characterized the 4 day timepoint, and represented 30.2, 28.6, and 4.8% (total 63.6%) abundance of the total 4D timepoint, but only represented 0.05, 0.5, and 0.03% (total 0.58%) abundance of the initial timepoint respectively. Other classifying Genera included *Viridibacillus*, *Bacillus*, *Staphylococcus*, *Streptococcus*, *Acinetobacter* and *Tepidibacter* which better characterized the initial timepoint (35.8%) compared to only 0.58% of the final timepoint.

**Table 2:** Most important classifying taxa for distinguishing early from late timepoints using Random Forest model

Most Important Classifying Taxa	Mean Decrease Gini	% of T0/T3	% of T4Day
*Viridibacillus*	9.10	6.9	0.003
*Sporosarcina*	8.27	0.05	30.2
*Peptoniphilus*	4.59	0.58	28.6
*Ignatzschineria*	4.50	0.03	4.8
*Bacillus*	2.13	4.4	0.24
*Staphylococcus*	1.86	5.6	0.0
*Streptococcus*	1.83	7.4	0.07
*Acinetobacter*	0.78	5.8	0.02
*Tepidibacter*	0.12	5.7	0.25
*Clostridium*	0.07	9.2	10.5

The identities of several potential pathogens and organisms with known high potential for resistance gene transfer or pathogenic potential were also identified in order to determine presence and detection over time (Table 3). We found that most pathogenic species were detected initially but were greatly reduced in the later timepoints with the exception of *Streptococcus sanguinis*, and *Clostridium botulinum* which increased as decomposition progressed.

**Table 3:** Assembled table of notable pathogenic species and their prevalence in early and late timepoints

Bacterial Species	Time 0	Time 4 Day	Human	Livestock
*Pseudomonas aeruginosa*	37	0	Y	Y
*Mycoplasma lipophilium*	7	23		
*Mycoplasma hyorhinisis*	202	1		Y
*Streptococcus agalactiae*	21	0	Y	Y
*Streptococcus dysgalactiae*	4137	2	Y	Y
*Streptococcus bovis*	13,431	125		Y
*Streptococcus sanguinis*	0	77	Y	
*Streptococcus suis*	6585	1		Y
*Actinobacillus pleuropheumoniae*	15,208	78		Y
*Leptospira licerisiae*	186	9		Y
*Parachlamydia acanthamoebae*	24	0	Y	Y
*Bacillus anthracis*	69	12	Y	Y
*Bacillus cereus*	1559	6	Y	
*Bacillus subtilis*	92	0	Y	
*Clostridium botulinum*	304	831	Y	Y
*Clostridium perfringens*	71,053	26,675	Y	
*Salmonella enterica*	352	0	Y	Y
*Francisella hispaniensis*	23	42	Y	
*Moraxella catarrhalis*	40	2	Y	
*Acinetobacter baumannii*	38,699	87	Y	
*Actinomyces hyovaginalis*	3033	16		Y
*Serratia marcescens*	117	20	Y	
*Enterococcus faecium*	101	5	Y	

## Discussion

The escalation of mass mortality events (the rapid die-offs of large numbers of organisms, MMEs), coupled with changes in global trends of increased zoonotic pathogen proliferation and antibiotic resistance, have led to increased attention and surveillance of these events to determine the impact of mass animal carcass input into ecosystems [13–15]. The fate of the carcasses is of great interest as temporal fluctuations in nutrient availability and biomass quantity could have substantial effects on microbial population growth rates and competitive interactions. These fluctuations could have long-standing effects on microbial community structure and function, altering decomposition rates and processes. Nutrient pulses can also become selective pressures by which evolutionary forces may act on other microbes and higher organisms. However, at present, the effects of MMEs or decomposition of increasing biomass on microbial community structure and function and individual physiology are poorly understood.

Our results showed no difference in microbial diversity with respect to biomass or scavenger exclusion by fencing indicating the resilience (*sensu* *lato*, [16]) of the associated microbial communities to be greater than the perturbation experienced (e.g., scavenging or mass). Of course, the inability to detect such an impact could be partially explained by the scale of our experiment [17]. As described, our sample size was limited thus preventing sufficient power for statistical detection of the perturbation impact on the associated microbial community. Furthermore, inclusion or exclusion of scavengers originating from the initial decomposition study of increasing biomass from the simulated MMEs may not have been robust enough to transfer to a subsequent death event. This will also require measurements across stages of decomposition (carcasses for this study were placed during advanced decay of initial decomposition study of increasing biomass from MMEs). Notwithstanding, methodology developed from this experiment allows us to engage in future experimentation with a more robust sample set and with increased initial biomass.

While we did not detect a difference with biomass or fencing treatments, there was a detectable difference in genera relative abundance between skin and ocular cavity communities after initial placement. This was expected in early timepoints as decomposition was not yet in active decay. Genera initially dominating the skin included those associated with soil or water, consistent with environmental exposure during the feral swine’s life. The ocular cavity communities, however, were dominated by organisms consistently found associated with oropharynx or other internal organs at 0 h, suggesting very little microbial transmigration across the cadaver landscape at this early timepoint. Skin and ocular cavity microbial communities did not differ significantly at the 3 h timepoint, suggestive of rapid transmigration between internal and external carcass-associated communities. Maggot (i.e., dipteran larvae) colonization and feeding behaviors could have also been a contributing factor to the comingling of microbial communities.

A decrease in Genera-level richness was detected in both skin and ocular cavity communities at the 4 day timepoint. However, there was a significant difference between the skin and ocular cavity communities at the 4 day timepoint, when active decay was apparent. Though both skin and ocular cavity communities had similar richness, they differed with relative abundance. Ocular cavity communities had an increase in *Clostridium* and *Peptoniphilus*, both anaerobic genera associated with human and animal digestive tracts [18]. Whereas, skin microbial communities had a higher abundance of *Sporosarcina* which are associated with soil communities [19].

Overall, skin communities showed higher richness when compared to ocular cavity communities and had a higher number of rare taxa at early timepoints. This richness decreased through the timepoints which could likely be due to out-competition for limited resources. Ocular cavity communities showed an increase in richness from the 0 h to the 3 h timepoint but were also decreased at the 4 day timepoint. Differences in richness of both anatomical locations likely reflect the sampling area as well as the environmental exposure of the skin. Shifts in relative abundance of ocular cavity communities could be a result of immune function cessation and microbial access to previously sterile spaces [20, 21], with dominant taxa possessing enzymes and other molecules to utilize these resources.

Environmental transitions generally entail a selective decrease in diversity because tenuous species will be outcompeted or die; this means that any given stress will select for adapted species [22]. Therefore, physiological attributes of each species within the microbial community will likely determine selection by the nutrient pulses. In most natural ecosystems, microorganisms are likely to experience alternating periods of unrestricted growth with surplus nutrients, nutrient-limited growth, and starvation. Changes in community structure as a result of nutrient fluctuations can occur because individual species differ in their uptake, storage or assimilation kinetics, and therefore have selective advantages at different points in the pulse cycle [7, 23, 24]. This has been shown in several studies of decomposition succession demonstrating enrichment of anaerobic communities over decomposition time [4, 25].

Analysis of the entire carcass communities by timepoint showed no significant differences within the 0 h and 3 h timepoints. However, there was a significant divergence at the 4 day timepoint. This is consistent with several other studies showing community stability during early decomposition. For instance, Pechal et al., and Burcham et al., demonstrated community stability in human and mouse necrobiomes for post-mortem intervals less than 48 h, but with divergence at later timepoints [7, 21]. Though mechanisms and reasons for this shift after this PMI are unclear, it is thought that waning immune cells and decomposition succession may be potential drivers [20]. Nevertheless, this consistency in stability may be useful for targeting taxa driving stability.

To further elucidate the potential effects of timepoint (i.e., decomposition succession) on microbiota composition, we determined the most important classifying taxa based on the relative abundances of OTUs at 0 h, 3 h, and 4 day. As expected, models lacked any discriminatory power between 0 h and 3 h. Predictive accuracy was higher between the combined early timepoints and 4 day. Genera with the highest importance scores for accurate prediction of timepoint generally overlapped with those exhibiting drastically depleted or increased relative abundances between early and late timepoints (Table 2).

The most discriminatory taxa for separating early timepoints were *Viridibacillus*, followed by *Bacillus*, *Staphylococcus*, *Acinetobacter*, and *Tepidibacter*. *Viridibacillus*, *Bacillus*, and *Acinetobacter* have been identified from soil samples, and are likely a reflection of host environment exposure ante-mortem. *Staphylococcus* is a normal commensal of skin and the nasopharynx. *Tepidibacter*, though not well characterized, is a thermophilic bacterium that has been characterized from soil and hydrothermal vents, but has also been identified, by next generation sequencing, from within human and other animal fecal and carcass microbiomes [26, 27].

*Sporosarcina*, *Peptoniphilus, Ignatzschineria* and *Clostridium* were the most important taxa for classifying the late timepoint (4 day). As previously mentioned, *Sporosarcina*, normal soil organisms, were found in higher abundance in the skin at early timepoints and likely transmigrated or were otherwise introduced into the carcass internal compartments to be a dominant species. *Peptoniphilus* and *Clostridium* are part of normal gut microflora and are anaerobic organisms; therefore, change from aerobic to anaerobic conditions at the late timepoint, among other factors within the carcass, was a likely driver for this shift.

Interestingly, *Ignatzschineria* was also found to be an important classifier for the late timepoint. This genera belongs to the family *Xanthomonadaceae*, class Gammaproteobacteria, and was first described in 2001 [28]. Species within this genus are anaerobic, and have been associated with maggots colonizing wounds, and also with blow flies and flesh flies colonizing carcasses [29–33]. We cannot rule out that these organisms were not present at the earlier timepoints, however if so, they fell below detection levels. Nevertheless, detection of *Ignatzschineria* at the 4 day timepoint suggests that insect associated microbes may influence decomposition associated microbial succession models. More work should be conducted with a focus on insect specific microbial taxa as well as spatial and temporal insect activity to determine whether other insect associated taxa may be dominant and important classifying taxa.

We also took advantage of our sample set to determine the prevalence and distribution of potential animal or zoonotic pathogens within our samples. Though not an exhaustive search, our sequencing analyses revealed sequence identities suggestive of pathogen presence in low abundance within our sample set. The majority of sequences were in higher abundance at our earliest timepoint, and detection decreased as decomposition progressed. From our limited search, only two of the sequence identities, *C. botulinum* and *S. sanguinis* increased over time, albeit only moderately. *C. botulinum* is an anaerobic organism that is most commonly isolated from soil or decaying vegetation, and produces toxins which may be ingested, leading to disease. *C. botulinum* has also been reported from swine intestinal samples, with concentrations varying by geographical location ([34] and references therein). The finding of these organisms associated with swine carcasses is likely due to their rooting behavior and diet. It is important to note that though we detected these organisms, the presence of these sequences is not indicative of viability or virulence, and further analyses would need to be undertaken to determine this, as well as how closely the identities here are to specific species functionally. Nevertheless, these findings do provide some insights into distribution of potential pathogens in feral swine populations. Based on our data, it can be inferred that feral swine could be a suitable reservoir for some pathogens. Additionally, although we measured the presence of soil-and/or animal-borne pathogens, a more comprehensive study with a larger sample size will be required to fully validate these findings. Based on the collective experience of the investigators involved in this study, a unified human and animal active and passive surveillance program is key to understanding the prevalence and distribution of the pathogens in humans and animals. The surveillance program would require a contemporary national and regional laboratory network system to detect and identify zoonotic diseases of public health importance.

Although results presented here are meaningful, this study was not without limitations. We purposefully chose to focus on microbial succession of secondary death events from a single carcass placed at the initial decomposition study sites for logistical feasibility, and to allow for more uniform comparisons across sites. But, as mentioned above, limited treatment replication and small sample size reduced the power for robust statistical analyses leading to less conclusive results. Additionally, the timeframe for studying decomposition and other ecological effects from a subsequent death event was only for four days. It would therefore be beneficial in future studies to include more study sites, and also feral swine at a non-mortality site, although no differences were observed with respect to differing biomass. Future large-scale studies investigating subsequent mortality events should also be expanded to include multiple decomposition stages, and a wider range of initial mortality biomass. Notwithstanding, data from this study aid in the understanding of structure of host-associated microbial species during decomposition to help predict ecosystem processes under higher-biomass mortality events.

## Conclusions

In conclusion, the goals of this study were to gain a broader understanding of the conserved effects of decomposition on microbial communities, and to establish the impact of various factors, including biomass, time, and initial invertebrate scavenger access on this natural progression. The applications from this study are many, ranging from establishing the environmental impacts of MMEs to understanding the importance of scavenger, and scavenger microbial community input on decomposition.

## Methods

### Initial mass mortality sites used for this study

Initial MMEs were constructed in wooded areas, predominantly loblolly pine, with a high percentage of tree cover, with an average daily temp of 30 °C (National Centers for Environmental Information). Each mass mortality site contained either 25, 180, or 725 kg of decomposing feral swine biomass, which was in advanced decay [35] at the time of swine placement for this study of subsequent death events. For each biomass there was one fenced and one unfenced replicate located at least 100 m apart to exclude large scavengers and to reduce the likelihood of microbial crossover from other sites, and aid in the isolation of invertebrate communities (Fig. 1) [8]. Fencing was constructed with rolled wire fencing surrounding an area of approximately 2 m^2^, and each plot was further covered with a black plastic mesh.

### Soil-exclusion experimental design

Feral swine used in this study, weighing approximately 22 kg each, were euthanized and frozen within two hours of death for 48 h before thawing at 23 °C. One feral swine was placed individually into open black plastic 121 Liter containers, that was then placed at one of six constructed mass mortality sites which were undergoing advanced decay, as described above (Fig. 1a). After 4 h of potential invertebrate exposure, containers were covered with mesh to prevent further insect contact and colonization and were transported to a common area (Fig. 1b).

### Sample collection

Skin and ocular cavity swab samples were collected from the swine. Ocular cavity swab samples were collected from ocular orbits in triplicate for each swine using sterile cotton swabs. Our rationale for collecting ocular cavity swab samples was that these spaces were more sterile than the skin and offered a more invasive sampling without puncturing the cadaver and thus compromising the natural decomposition process [36]. Skin swab samples were collected by vigorously rubbing the swabs over three random skin locations for one minute. Collected samples were stored in separate microcentrifuge tubes containing 0.5 ml RNAlater (Invitrogen, Waltham MA). Samples were collected at 0 h, 3 h, and 4 days after initial placement at the MME sites and were stored at 4 °C until analyses.

### DNA extraction

DNA was extracted from samples using TRIzol phenol-chloroform extraction protocol and purified using a PowerClean DNA clean-up kit (Qiagen, Germantown MD) according to manufacturer’s instructions. Resulting DNA concentration was quantified using a Qubit 2.0. DNA was amplified by PCR using primers targeting the V4 region of 16S rRNA (515F-806R) and methodology as previously described for the Earth Microbiome Project [37]. Samples were sent to Michigan State University Genomics Core Facility for 16 s rRNA sequencing targeting the V4 region.

### 16 s sequence processing

Raw Illumina-barcoded FASTQ files for 16S rRNA paired-end reads were assembled, quality-filtered, demultiplexed, and analyzed with QIIME using default settings [38]. Sequencing yielded an average of 100,000 reads per sample (min 17,000) and included almost 850 genus-level taxa. OTUs were assigned using the Greengenes database [39]. One sample was removed due to low-yield (fewer than 1000 reads), and samples were rarefied to 17,000 reads which removed 27 singleton OTUs. Microbial community analyses were then performed for Phylum and Genus-level taxa.

### Statistical analyses

All statistical analyses were performed using the phyloseq 3.8 and vegan 2.4–4 packages in R [40, 41]. For the purposes of statistical robustness, fenced and unfenced samples were analyzed as replicates because no significant difference in microbial community structure was found (Table 1). Microbial community richness and evenness were evaluated using Chao richness and Shannon-Weaver alpha diversity [2, 42]. Differences in microbial populations at Genus level were evaluated using Bray-Curtis beta diversity [43]. PERMANOVA analyses were used to compare main and interactive effects using the adonis function of the vegan 2.0–7 package in R, according to McCune and Grace [44]. A Random Forest algorithm was used to identify the highest-ranking predictor taxa among timepoints using highest mean decrease gini scores. This analysis was performed using the randomForest R package using default parameters (1000 trees) [2, 45–47]. This model works by determining how much a model decreases in accuracy by removing a certain taxon [48].

### Potential pathogen sequence detection and analyses

The list of assigned species OTUs were referenced against literature sources of known human and animal-associated pathogens. Common pathogens, and those in highest abundance were identified and compiled. Those with fewer than 10 sequence reads or rare-exception literature cases were excluded from the results table.

## Data Availability

The datasets used and/or analyzed during the current study are available from the corresponding author on reasonable request.

## Abbreviations

MME: Mass mortality event
PCR: Polymerase chain reaction
OTU: Operational Taxonomic Unit
PERMANOVA: Permutational multivariate analysis of variance
